# Intraductal Papillary Mucinous Neoplasms of the Pancreas: A Review of Their Genetic Characteristics and Mouse Models

**DOI:** 10.3390/cancers13215296

**Published:** 2021-10-22

**Authors:** Jin Li, Tao Wei, Jian Zhang, Tingbo Liang

**Affiliations:** 1 Department of Hepatobiliary and Pancreatic Surgery, School of Medicine, The First Affiliated Hospital of Zhejiang University, 79 Qingchun Road, Hangzhou 310003, China; lijinlj@zju.edu.cn (J.L.); wei_tao@zju.edu.cn (T.W.); johnzhang@zju.edu.cn (J.Z.); 2 Zhejiang Provincial Key Laboratory of Pancreatic Disease, Hangzhou 310000, China; 3 Innovation Center for the Study of Pancreatic Diseases, Hangzhou 310000, China; 4 Zhejiang Provincial Clinical Research Center for the Study of Hepatobiliary & Pancreatic Diseases, Hangzhou 310000, China; 5 Cancer Center, Zhejiang University, Hangzhou 310058, China

**Keywords:** IPMN, genetic mouse models, molecular signature, pathogenesis, pancreatic cancer

## Abstract

**Simple Summary:**

Pancreatic cancer is one of the deadliest cancers with the lowest survival rate. Little progress has been achieved in prolonging the survival for patients with pancreatic adenocarcinoma. Hence, special attention should be paid to pre-cancerous lesions, for instance, an intraductal papillary mucinous neoplasm (IPMN). Here, we reviewed its genetic characteristics and the mouse models involving mutations in specific pathways, and updated our current perception of how this lesion develops into a precursor of invasive cancer.

**Abstract:**

The intraductal papillary mucinous neoplasm (IPMN) is attracting research attention because of its increasing incidence and proven potential to progress into invasive pancreatic ductal adenocarcinoma (PDAC). In this review, we summarized the key signaling pathways or protein complexes (GPCR, TGF, SWI/SNF, WNT, and PI3K) that appear to be involved in IPMN pathogenesis. In addition, we collected information regarding all the genetic mouse models that mimic the human IPMN phenotype with specific immunohistochemistry techniques. The mouse models enable us to gain insight into the complex mechanism of the origin of IPMN, revealing that it can be developed from both acinar cells and duct cells according to different models. Furthermore, recent genomic studies describe the potential mechanism by which heterogeneous IPMN gives rise to malignant carcinoma through sequential, branch-off, or de novo approaches. The most intractable problem is that the risk of malignancy persists to some extent even if the primary IPMN is excised with a perfect margin, calling for the re-evaluation and improvement of diagnostic, pre-emptive, and therapeutic measures.

## 1. Introduction

Pancreatic cysts are being identified with increasing frequency as a result of the increasing use of cross-sectional imaging [1], the enhanced quality of imaging-workup, and the aging population. The incidence of pancreatic cysts increases with age, with a prevalence of 0.5% in those younger than 40 and 25% in those in their 70s [2,3]. However, these incidences are likely to be underestimated because pancreatic cysts often present with no clinical symptoms and the lesion is frequently detected serendipitously using abdominal imaging performed for another condition or during regular check-ups.

Pancreatic cystic tumors are a large family consisting of the following major groups: serous tumors (including serous cyst adenoma and cystadenocarcinoma); solid pseudopapillary neoplasms (SPNs); pancreatic pseudocysts; and cystic neuroendocrine tumors (cNETs) and mucinous tumors, including mucinous cystic neoplasia (MCNs) and intraductal papillary mucinous neoplasia (IPMNs) [4]. Among them, IPMN is the most common, accounting for about 20–40% of all pancreatic cystic tumors and for 1–3% of all exocrine pancreatic neoplasms [5]. The mean age of the diagnosis of IPMN is at the mid-60s equally in men and women [6,7]. IPMNs have been reported to be more common in patients who smoke cigarettes [8], have diabetes, have Peutz–Jeghers syndrome, have familial adenomatous polyposis syndrome [9], or familial pancreatic carcinoma, or who have a family history of pancreatic ductal adenocarcinoma [10]. IPMN has become a research hotspot because of its precursor role in invasive pancreatic ductal adenocarcinoma (PDAC) [11], together with two other precursors, namely pancreatic intraepithelial neoplasia (PanINs) and MCN [12].

IPMNs are defined as potentially malignant intraductal epithelial neoplasms that are grossly visible [13] (classical standard of > 10 mm, with a lower cutoff of 5 mm as acknowledged recently [14,15,16]), which consist of mucin-producing columnar cells. IPMNs can be classified morphologically as the main duct (MD), branch duct (BD), or both, according to their location [13,17]. The molecular mechanism of PanIN is relatively well understood, whereas that of IPMN is not. In this review, we summarize the recent advances in the study of IPMN pathogenesis, with particular emphasis on genomic profiling and on the use of genetically engineered mouse models (GEMMs).

## 2. Diagnosis and Clinical Pathology

The diagnosis of pancreatic cystic neoplasms typically starts with cross-sectional imaging, i.e., computed tomography (CT) and magnetic resonance cholangio-pancreatography (MRCP). Evaluation of the communication between the dilated branch ducts and the main pancreatic duct is important for distinguishing IPMNs from other cystic lesions [11]. For patients who need additional evaluation, endoscopic ultrasound with fine-needle aspiration (EUS-FNA) can provide high-quality imaging of the pancreas and the opportunity to sample pancreatic lesions for both cytology and cyst fluid analysis (amylase, carcinoembryonic antigen [CEA] level) [18]. If there is still concern about possible malignancy after EUS-FNA or if the extent of the IPMN is unclear, additional testing may be done. Testing may include endoscopic retrograde cholangio-pancreatography (ERCP), with aspiration of the pancreatic duct contents or brushing of the pancreatic duct, pancreatoscopy, intraductal ultrasonography, positron emission tomography (PET), or assessment of serum tumor markers.

The reported risk of malignancy for patients with MD-IPMN ranges from 38% to 68%, but is much lower for BD-IPMN, which ranges from 11% to 30% in patients who received resection [15,19,20]. 

The 2015 American Gastroenterological Association (AGA) guidelines do not recommend resection for main duct dilatation alone, unless it involves the presence of a nodule or is cytologically positive for malignancy [21]. However, the 2017 International Association of Pancreatology (IAP) [22] and 2018 European [23] guidelines are more radical. Patients with the involvement of the main pancreatic duct (≥10 mm) are advised to undergo surgical resection. 

However, for BD-IPMN, the situation is more complicated. According to the 2018 European guidelines, the absolute indications for resection (high-risk stigmata) include: the presence of obstructive jaundice, an enhanced mural nodule (≥5 mm) or solid mass, and cytology positive for high-grade dysplasia or cancer [23]. In addition, conservative treatment seems to be appropriate, with a high 5-year-disease-specific survival (DSS) of 96% in elderly people who only have worrisome features [24]. Notably, current clinical criteria are not precise enough to recognize the risk and molecular markers are urgently required [25]. 

Histologically, BD-IPMN almost always corresponds to a gastric type and MD-IPMN can be further divided into intestinal, oncocytic, and pancreatobiliary types. These four subtypes can also be distinguished through immunohistochemical staining of mucin 1 (MUC1); MUC2; mucin 5AC, oligomeric mucus/gel-forming (MUC5AC); and caudal type homeobox 2 (CDX2; Table 1) [26]. 

The gastric type is the most common, with a favorable prognosis and lower malignant risk [30]. The oncocytic type shows the same MUC levels as the pancreatobiliary type but the lining cells reveal strong eosinophilic cytoplasm [28]. This histological classification is widely leveraged among the construction of GEMMs because each subtype might correspond to a certain mechanism. 

## 3. Genetic Signatures and Their Clinical Application

Although IPMN shares some common genetic mutations with pancreatic infiltrating ductal adenocarcinoma (e.g., KRAS proto-oncogene, GTPase (KRAS), and tumor protein P53 (TP53) and cyclin-dependent kinase inhibitor 2A (CDKN2A)), it additionally comprises unique mutations in the guanine nucleotide-binding protein-stimulating α subunit (GNAS; 41–79%) [31,32,33] and ubiquitin E3 ligase ring finger 43 (RNF43; 38%) [34,35], which are putatively related to the pathogenesis of IPMN. Basturk et al. performed comprehensive molecular sequencing to identify the mutation frequency of other genes, namely chromatin-remodeling genes (32%), *PI3K* (encoding phosphatidylinositol-4,5 -bisphosphate 3-kinase) (27%), and *FGFR2* (encoding fibroblast growth factor receptor 2) (18%) [35].

*GNAS* [36] serves as a key oncogene in IPMN. The oncogenic mutations of GNAS (primarily R201C and R201H) markedly activate adenylyl cyclase, leading to the intensified formation of cyclic AMP, a second messenger that stimulates multiple downstream effectors [37]. GNAS mutations seem to be specific for IPMNs [38] and are most prevalent in intestinal-type IPMNs, being found in 78–100% of these neoplasms; however, *Gnas* mutations only mimic gastric or pancreatobiliary type IPMN in GEMMs, which remains a puzzle [39,40]. 

RNF43 is acknowledged as a tumor suppressor and a negative regulator of the Wnt pathway by reducing the membrane level of the Frizzled receptor [41]. Unlike *Gnas* mutations, the knockout or mutation of *Rnf43* only accelerates Kras-driven tumorigenesis [42] and has not been verified to simulate IPMN lesions in mouse models. These mutation features might be utilized in molecular-targeted therapy, especially for RNF43, where antibodies targeting the Wnt pathway have already been applied. 

Recently, several studies showed distinct genetic features in different stages of IPMN. Hotspot mutation of *KLF4* (encoding Kruppel such as factor 4) in tissue samples was recently revealed using multiregion whole-exome sequencing, which has been proven to be enriched especially in low-grade dysplasia [43]. In addition, *MUC5AC* expression in circulating extracellular vesicles was significantly higher (sensitivity of 82%, specificity of 100%) in high-grade lesions [44]. Genetic heterogeneity of early driver genes is significantly more prevalent in low-grade IPMNs [45].

According to the genomic characteristics of IPMN, many emerging diagnostic tools have been tested using advanced sequencing technology for the early detection and surveillance of invasive progression. The basic logic is to detect the unique early mutation of *GNAS* and *TP53*/*SMAD4* (encoding SMAD family member 4) mutations, which are related to high-grade dysplasia [33]. 

Pancreatic cyst fluid obtained through EUS-FNA [46] is a reliable sample for IPMN diagnosis (sensitivity, 89%; specificity, 100%) [47], with a low risk of complications (2–3%) [23]. Moreover, the monoclonal antibody Das-1 in the cyst fluid can be analyzed to define the risk of malignancy (88% sensitivity, 99% specificity) [48]. A study also used secretin-stimulated pancreatic fluid to identify IPMN through *GNAS* mutations (64.1% sensitivity, 100% specificity) [49]. In addition, DNA methylation can also be analyzed for molecular diagnosis (sensitivity, 83%; specificity, 86%) [50]. Targeted genotyping (*GNAS*/*KRAS*) of cell-free DNA (cfDNA) from blood samples can also distinguish IPMN from control cases (sensitivity, 81%; specificity, 84.2%) and the amount of cfDNA detected is much higher in metastatic pancreatic ductal adenocarcinoma (PDAC) than in IPMN for differential diagnosis [51]. Considering the higher abundance of circulating tumor cells (CTCs) in portal vein blood compared to in peripheral blood, researchers have discovered that the count of mesenchymal-CTCs and vimentin+ CTCs correlates with a poorer differentiated tumor and a shorter survival [52,53].

For malignancy detection, *SMAD4*/*TP53* mutation detected in pancreatic fluid can distinguish PDAC from IPMN cases, with a 32.4% sensitivity and 100% specificity [54]. Another study reported a new method to detect circulating pancreas epithelial cells in blood samples before tumor formation [55] because epithelial denudation exists widely in pre-cancerous cases [56].

## 4. The Progression of IPMN into PDAC

It used to be taken for granted that different IPMNs found in a patient have monoclonal origins [57] and are direct precursors to pancreatic cancer [58]; however, more recent studies indicate the opposite. Single-cell sequencing [59] and microdissection, followed by capture-based targeted sequencing [45], were performed on IPMN samples, which suggested polyclonal precursors and revealed distinct mutations in driver genes.

Invasive adenocarcinoma found in a patient with concurrent IPMN does not always originate from the cystic lesion. Felsenstein et al. collected samples comprising IPMN and adenocarcinoma simultaneously, and isolated them for targeted next generation-sequencing, which revealed a striking result: 18% of adenocarcinomas were actually independent of the concurrent IPMN [60]. Further studies proposed three pathways of cancerization, classified as sequential, branch-off, and de novo, with a frequency of about 1/3 each [38,61]. This recent genomic evidence suggests that IPMN should no longer be considered as a single locoregional disease but rather is a complex lesion with genetic heterogeneity, which induces the carcinogenesis of the whole pancreas. 

**Figure 1:**
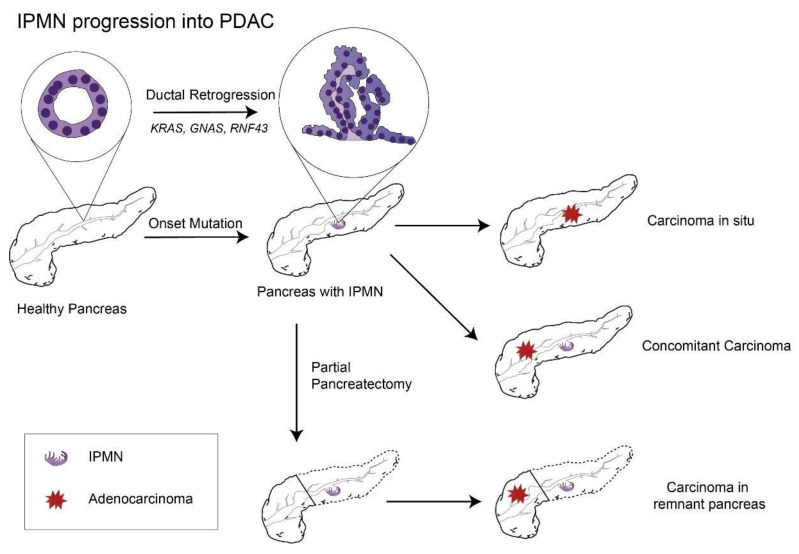
The remaining risks of PDAC progression in the remnant pancreas after partial pancreatectomy to remove the primary IPMN lesion. Abbreviations: IPMN, intraductal papillary mucinous neoplasm; PDAC, pancreatic ductal adenocarcinoma; GNAS, guanine nucleotide-binding protein-stimulating α subunit; and RNF43: ubiquitin E3 ligase ring finger 43.

One meta study, which included both MD-IPMN and BD-IPMN cases followed without surgery, analyzed the cumulative incidence of pancreatic cancer and found that the 10-year chance of developing pancreatic cancer was 25% for MD-IPMN [62]. Although the risk of cancer might decrease greatly after partial pancreatectomy of MD-IPMN with a negative margin, it still exists within the remnant pancreas [63] (with a 10-year incidence of pancreatic cancer of 38.3% for high-grade dysplasia, 3.0% for low-grade dysplasia, and 21.2% in total [64]), suggesting that IPMN is a sign that the whole pancreas is undergoing an irreversible process of carcinogenesis (Figure 1). 

For BD-IPMN, retrospective studies discovered that the overall cumulative incidence rates of pancreatic carcinoma were 1.1% at 5 years, 3.5% at 10 years, and 12.0% at 15 years, and notably, the incidence of IPMN-derived carcinoma and concomitant PDAC was almost equal [65,66]. Therefore, current guidelines for BD-IPMN are rather conservative [20]. The risk factors of malignancy at follow-up involves cyst size (≥30 mm), increased serum level of carbohydrate antigen 19-9 (CA19-9), and thickened cyst walls [22]. In addition, a growth rate of ≥2.5 mm/y is recommended as an independent predictor of pancreatic cancer during surveillance. 

To date, surgery is the only effective method to eradicate IPMN. The choices include total pancreatectomy, pancreaticoduodenectomy, distal pancreatectomy, and segmental resection of the tumor, which is determined by the location of the tumor and the extent of involvement of the gland [20]. 

There are alternatives that are still in the experimental phase. EUS-guided pancreatic cyst ablation using ethanol and/or paclitaxel [67], and SB-IPMN enucleation using a combination of blunt dissection, bipolar cautery, small clips, and/or fine sutures [68] are two novel approaches that have been tested in several clinical trials. Both modalities enable a shorter operative time and in-hospital stay, and, importantly, a better reserved endocrine and exocrine function [69]. However, long-term safety issues have not been well investigated. Additionally the ablation is reported to have a 2–10% rate of complications, presumably linked to the use of ethanol [70,71]. 

Considering that the rate of postoperative severe complications was determined as 14.0% [72], we still need more biomarkers to better predict the malignant risks for further decisions concerning surgery. 

New therapies involving molecular treatment are also urgently required. However, a selection bias of the patients cannot be ignored. For example, patients left unresected when diagnosed with MD-IPMN (pancreatic duct size of ≥ 10 mm) is against the current IAP and European guidelines, unless there are concerns related to the patient’s poor physical condition. 

## 5. Genetically Engineered Mouse Models of IPMN

Over the past three decades, with the increasing understanding of the genetic mutations underlying tumorigenesis, researchers have developed various GEMMs that reproduce the genetic events in in vivo settings, allowing for de novo tumor formation in a native immune-proficient microenvironment [73]. GEMMs have provided a platform to define genotype–phenotype relationships in IPMN pathobiology and have greatly increased our understanding of its pathological progress. Almost every PDAC harbors oncogenic mutations in the *KRAS* gene [74], the protein product of which mediates a wide variety of cellular functions, including proliferation, differentiation, and survival. A widely used classical mouse model to elicit mPanIN that can further progress to PDAC (latency of >1 y) [75] is based on the KRAS mutation, which strongly stimulates its intrinsically inefficient activity [76]. 

Although the sole mutation of KRAS is not strong enough to induce IPMN lesions in mice, artificially generating another alteration of a certain gene in the meantime has proven to be valid. Therefore, there is a general strategy to create IPMN models by using the Lox-stop-lox *Kras*G12D (*LSL-Kras*G12D) allele [77], which undergoes Cre-mediated excision of the stop codon concomitant with another mutation, deletion, or overexpression. 

By manipulating different promoters that express in different cell types or at different time points (prenatal or postnatal), researchers have attributed the pivotal role in the formation of mouse IPMN to pancreatic duct cells, as is often verified in human IPMN. The main mechanism is “ductal retrogression” at the onset of IPMN, in which the mature duct cells downregulate ductal markers such as SRY box 9 (SOX9) and present a morphology more similar to progenitor cells [78]. 

We have collected all the genetic mouse models of IPMN published since 2006 (when the first model was created by Bardeesy et al.) as well as those that provide an adequate description, including the histological types, invasion rates, targeted cell type (*P48* is another name for *Ptf1a*, a promoter targeting multipotent progenitors of pancreatic ducts and of both exocrine and endocrine cells during embryologic development, which is activated at E9.5 and retained in acinar cells [79,80,81]), and metastasis (if available). We summarized the involved signal pathways of the potential mechanisms (Table 2). The existing models provide us with inspirations and insights to build more mouse models concentrating on other genes involved in these pathways or complexes, i.e., G protein-coupled receptors (GPCR) and transforming growth factors (TGFs), as well as the SWI/SNF (SWItch/sucrose non-fermentable), WNT, and PI3K pathways.

(1) Concerning the GPCR pathway: GNAS is a component of GPCR-regulated adenylyl cyclase signal transduction pathways. *Gnas* mutations have been applied in mouse models, with mutations R201H or R201C being feasible for the formation of IPMN [82,83]. However, there remains some discrepancies between human IPMN and these established mouse models. The transgenic mouse models needed to have synergistic mutations of *Gnas* and *Kras* to develop a cystic tumor, while in human cases, IPMNs can develop with mutations in either *GNAS* or *KRA*S. In addition, the cystic tumor developed in the *Tg-Gnas*R201H*:Kras*G12D mice always showed a gastric or pancreatobiliary phenotype.
(2) Concerning the TGF-β pathway: TGF-β is a secreted polypeptide that can bind to its receptors and trigger phosphorylation of SMAD2 and SMAD3. Phosphorylated SMAD2 and SMAD3 then interact with SMAD4. The SMAD2/3/4 complex accumulates within the nucleus and acts as a potent inhibitor of epithelial cell growth and survival via modulation of the expression of cell cycle regulators and the activation of apoptosis [84]. Paradoxically, TGF-β is known to be a growth suppressor in the non-neoplastic epithelium but acts as a metastatic tumor promoter in advanced cancers [85,86], thus it might play a key role in regulating epithelium identity.
(3) Since Bardeesy et al. discovered that disturbance of TGF-β/SMAD4-signaling induces the formation of IPMN and progression of PDAC, other targets in this superfamily have been associated with cyst formation. The deletion of the genes such as *Acvr1b* (encoding activin A receptor type 1B), *Tif1g* (encoding transcription intermediary factor 1-gamma, which regulates SMAD4), and Tff2 (encoding trefoil Factor 2, an upstream element of SMAD4) in pancreas progenitor cells, and their cooperation with *KrasG12D* have been proven to induce IPMNs.
(4) Concerning the SWI/SNF complex: Mutations of the SWI/SNF complex subunit genes have been found in 12–23% of human PDAC cases and reduced or lost expression of *BRG1* (encoding Brahma protein-like 1) was observed in human IPMN [87]. Among the chromatin-remodeling complexes, homozygous deletion of *Brg1* or *Arid1a* (encoding AT-rich interaction domain 1A) has been proved to elicit IPMN lesions in mouse models. A recent study showed that these two genes cooperate to inhibit the dedifferentiation of duct cells and the subsequent IPMN formation through the regulation of genes that sustain pancreatic duct cell identity, including *Sox9* [78].

Unlike other genes that have similar consequences in the development of PanIN and IPMN, SWI/SNF subunit genes show complex expression in different cell types and at different time points [88,89]. *Brg1* or *Arid1a* deletion inhibits KRAS-dependent acinar-to-ductal metaplasia (ADM) and PanIN development of acinar cells, but promotes the preneoplastic transformation in duct cells. Spontaneous PanIN formation is drastically attenuated in the *Brg*1 model [90] but can still be seen in the *Arid1a* model [91,92]. In addition, the malignant risk is higher and the cancer progresses faster in the *Brg1* (3/7 at 9 w) knockout model than in the *Arid1a* knockout model (3/15 at 48 w), suggesting a more important role of *Brg1* than *Arid1a* in invasive IPMN. 

Furthermore, *Ptf1a*-*Cre*ERT2 (in which *Ptf1a* encodes pancreas-associated transcription factor 1a and *Cre*ERT2 is Cre recombinase (Cre) fused to a mutant estrogen ligand-binding domain), KrasG12D, and *Arid1a*f/f mice treated with tamoxifen (a postnatal Acini-targeting model) developed PanINs but not IPMNs, while *Hnf1b*-*Cre*ERT2, KrasG12D, and *Arid1a*f/f (a postnatal ductal cell-targeting model, in which *Hnf1b* encodes hepatocyte nuclear factor 1 beta) mice developed cystic lesions whose mucin expression pattern was similar to the IPMN in *Ptf1a*-*Cre*, KrasG12D, and *Arid1a*f/f mice. Therefore, unlike the *Tff2*^−/−^ model, in which the pancreatic duct gland cells are believed to be the cellular origin, mouse *Arid1a* or *Brg1*-deficient IPMNs have been proven to originate from the ductal compartment rather than from the acinar compartment [91].

(5) Concerning the PI3K pathway: Loss of *PTEN* (encoding phosphatase and the tensin homolog, also known as phosphatidylinositol-3,4,5-trisphosphate 3-phosphatase) expression occurs in human PDAC and is associated with poor prognosis of IPMN [93]. Combined *Pten* deletion and expression of oncogenic *Kras* in embryonic pancreatic precursor cells with *Pdx1-Cre* (in which *Pdx1* encodes pancreatic and duodenal homeobox 1) failed to induce IPMN [94,95]. However, Kopp et al. found that IPMN only formed in response to postnatal ductal cell-specific, but not acinar cell-specific, *Pten* deletion [1]. This postnatal model can better mimic human IPMNs, which are usually solitary, and the lesions tend to occur in mature and highly differentiated cells rather than in progenitor cells. The postnatal homozygous deletion of *Pten* alone is able to generate IPMN, with faster progression to PDAC when combined with *Kras* mutations.
(6) Concerning others: *STK11* is a tumor suppressor gene that encodes serine–threonine kinase 11 (also known as liver kinase B1 (LKB1)), which is central to the control of cellular energy metabolism. Patients with heterozygous germline *LKB1* mutations (i.e., patients with Peutz–Jeghers syndrome) show an elevated incidence of IPMN [96]. Collet et al. generated a new model driving IPMN formation from well-identified postnatal duct cells, termed tamoxifen-induced *Sox9-Cre*ER, *LSL-Kras*G12D, and *Lkb1f/f* [97], in which the loss of the LKB1 function was proven to suppress Wnt-signaling to generate IPMNs [98].

Another IPMN mouse model comprising the transgenic overexpression of *Tgfα* under the control of the pancreatic Elastase promoter (*Ela-Tgfα*), combined with the KRAS G12D mutation, is highly metastatic, with the rate of 50% at 6–8 months, predominantly in liver, lung, peritoneum, and lymph nodes [26]. This might suggest that the downstream epidermal growth factor receptor (EGFR)/signal transducer and activator of transcription 3 (STAT3)-signaling is critical for IPMN and disseminated metastases.

**Table 2:** Genetically engineered mouse models of IPMN.

GEM Models	Histological Type	Latency	Invasive	Targeted Cell Type [99]	Function	Metastases	References
*Ptf1a-cre; LSL-Kras*^G12D^; and CAG*-LSL-Gnas^R201H;^*	Gastric and pancreatobiliary	4–5 w	NA	Acinar, duct, and endocrine	GPCR	Die at 5–6 w	Taki et al., 2016 [82]
*P48-Cre;LSL-Kras*^G12D^ and *Rosa26R-LSL-rtTA-* *Tet-OGnas^R201C^*	Pancreatobiliary	10 w	29% at 43 w	Acinar, duct, and endocrine	GPCR	20%	Ideno et al., 2018 [83]
*Ptf1a-Cre; LSL-Kras*^G12D^; and *Smad4^f/f^*	Gastric	8 w	16.7%	Acinar, duct, and endocrine	TGFβ	NA	Bardeesy et al., 2006 [100]
*Pdx1-Cre; LSL-Kras*^G12D^*;* and *Tif1γ^f/f^*	NA	7 w	0 at 13 w	Acinar, duct, and endocrine	TGFβ	NA	Vincent et al., 2009 [101] Vincent et al., 2012 [102]
*Pdx1-Cre; LSL-Kras*^G12D^*;* and *Acvr1b^f/f^*	NA	12 w	72% at 3–9 m	Acinar, duct, and endocrine	TGFβ	9%	Qiu et al., 2016 [103]
*Pdx1-Cre; LSL-Kras*^G12D^; and *Tff2^−/−^*	Gastric	6 w	16.7%	Acinar, duct, and endocrine	TGFβ	16.7%	Yamaguchi et al., 2016 [104]
*Ptf1a-Cre; LSL-Kras*^G12D^*;* and *Brg1^f/f^*	Pancreatobiliary	9 w	43% at 9 w, 71% at 18 w	Acinar, duct, and endocrine	SWI/SNF	NA	Von Figura et al., 2014 [105]
*Ptf1a-Cre; Kras*^G12D^*;* and *Arid1a^f/f^*	Gastric pancreatobiliary and oncocytic	12 w	20% at 48 w	Acinar, duct, and endocrine	SWI/SNF	3/19	Wenjia Wang et al., 2019 [92] Kimura et al., 2018 [91]
*Sox9-Cre*^ERT2^ and *Pten^f/f^*	Pancreatobiliary and oncocytic	6–14 m	31.5%	Duct	PI3K pathway	NA	Kopp et al., 2018 [1]
*Sox9-Cr*e^ERT2^*; LSL-Kras*^G12D^*;* and *Pten^f/+^*	Mainly pancreatobiliary	4–8 m	70%	Duct	PI3K pathway	NA	Kopp et al., 2018 [1]
*Sox9-Cre*^ER^*; LSL-Kras*^G12D^*;* and *Lkb1^f/f^*	Gastric	8 w	Yes	Duct	WNT/β-cat	NA	Collet et. al, 2019 [97]
*P48-Cre; LSL- Kras*^G12D^*;* and *Ela-Tgfa*	Pancreatobiliary	12 w	Died at 7 m	Acinar, duct, and endocrine	TGFa/EGFR	50%	Siveke et al., 2007 [26]

Abbreviations: GEM, genetically engineered mouse models; N/A, not available; GPCR, G protein-coupled receptors; TGFs, transforming growth factors; SWI/SNF, SWItch/sucrose non-fermentable; w, weeks; Lkb1, liver kinase B1; PTEN, phosphatase and tensin homolog; EGFR, epidermal growth factor receptor; SOX9, SRY box 9; LSL, Lox-stop-lox; Arid1a, AT-rich interaction domain 1A; Brg1, encoding Brahma protein-like 1; Tff2, trefoil Factor 2; Acvr1b, activin A receptor type 1B; Tif1g, transcription intermediary factor 1-gamma; Ela, elastase; and Gnas, guanine nucleotide-binding protein-stimulating α subunit.

## 6. Conclusions and Future Perspectives

PDAC is the fourth leading cause of cancer-related death in the United States, with an approximately 9% five-year survival rate [106]. Little progress has been achieved in prolonging the survival for patients with pancreatic adenocarcinoma. Hence, special attention should be paid to pre-cancerous lesions, for instance, IPMNs.

We summarized key signal pathways or complexes (GPCR, TGF, SWI/SNF, WNT, and PI3K) in IPMN pathogenesis, which are able to elicit IPMN lesions in genetic mouse models. Many other effector genes involved in these pathways might have the potential to generate similar IPMN lesions in mice, which needs to be further tested in the future. Several recent mouse models targeting postnatal duct cells with the induction of tamoxifen provided evidence for the ductal origin of IPMN. These various murine models can serve as a preclinical platform to address prevailing questions, from the characterization and dissection of both histopathological and molecular features to the response to novel therapies. 

However, pre-clinical mouse models have some intrinsic limitations. The genetic models are almost all based on bacteriophage-derived Cre recombinase, which means the oncogene activation and target ablation occur at the same time and in the same cells [99]. In addition, human IPMNs often display a dozen altered signal pathways, which cannot be attributed to one single gene. Among all the mouse models, the intestinal type of IPMN, which specifically expresses MUC2 and CDX2 [64], has not been successfully mimicked, representing a knowledge gap regarding the pathogenesis of the intestinal pathway. Future studies may have to focus on the exploration of mutation signatures in the intestinal type to identify the pivotal driver genes. It is important to note that this study only includes the mouse models based on the technology of the Cre recombinase system and there are other strategies, such as oncogenes [107] or the CRISPR/Cas9 [108,109] delivery-based system in the context of in vivo electroporation technology, to induce murine pancreatic neoplasms. This preclinical model is promising in developing more IPMN mouse models in the near future.

Although pancreatic surgery is the only effective method to treat IPMN, the malignant risk only decreases to a slight extent, leaving the remnant pancreas at high risk. Recent molecular findings based on next generation-sequencing indicated that IPMN is often heterogeneous and complex, and might generate adenocarcinoma inside or from a distant position that seems to be intact. Therefore, we propose a new concept: IPMN is not only a traditional lesion in situ but also represents dispersive damage, including changes to immune cells, cytokines, and stroma cells [110,111], which favor carcinogenesis whether the original IPMN is excised or not. Consequently, traditional therapy, such as surgery, is not sufficient when confronted with this intractable disease and more molecular therapies need to be developed to supplement surgery, given that adjuvant therapy for invasive IPMN has proven to be efficient in improving overall survival [112].

In conclusion, we reviewed the existing literature about IPMN, summarized its genetic characteristics and the mouse models involving mutations in specific pathways, and updated our current perception of how IPMN develops into a precursor of PDAC. 

## Figures and Tables

**Table 1:** Description of four historical types of IPMN.

Historical Type	Location	Morphology	MUC Expression [27]				Frequency [28]	Prognosis	Associated Carcinoma
			MUC1	MUC2	MUC5AC	CDX2			
Gastric	Branch duct	Thick finger-like papillae	–	–	+	–	49–70	Favorable	Tubular adenocarcinoma
Intestinal	Main duct	Villous papillae	–	+	+	+	20–35	Favorable	Colloid carcinomas
Pancreatobiliary	Main duct	Complex thin-branching papillae	+	–	+	–	7	Poor	Tubular adenocarcinoma
Oncocytic	Main duct	Complex thick-branching papillae	+	–	+	–	3–8	Poor	Oncocytic carcinoma [29]

Abbreviations: IPMN, intraductal papillary mucinous neoplasm; MUC, mucin; MUC5AC, mucin 5AC, oligomeric mucus/gel-forming; and CDX2: caudal type homeobox 2.

## Data Availability

Not applicable.
